# Impact of a “Pharmacist First” innovative workflow plan in patients with hypertension and/or diabetes

**DOI:** 10.1177/17151635211016498

**Published:** 2021-05-27

**Authors:** Randy So, Yazid N. Al Hamarneh, Carlene Oleksyn, Mary Purschke, Ross T. Tsuyuki

**Affiliations:** University of Alberta, Edmonton; University of Alberta, Edmonton; Mint Health + Drugs: Meridian, Stony Plain; Mainstreet Home Health Pharmacy, Stony Plain, Alberta; University of Alberta, Edmonton

## Abstract

Usual community pharmacy workflow, whereby patients might see a pharmacist at the end of the dispensing process, is not conducive to proactive patient-centred care. The objective of this study was to evaluate the impact of the “Pharmacist First” (P1st) workflow model on blood pressure and glycemic control in patients with hypertension and/or diabetes. This retrospective review was set in 2 community pharmacies that use the P1st model in the Greater Edmonton Region. The population entailed patients with hypertension and/or type 1 or 2 diabetes who received care via the P1st workflow model. The P1st workflow model places the patient in immediate contact with the pharmacist. The pharmacist first assesses prescription appropriateness, reviews relevant laboratory tests, discusses chronic disease control and addresses any questions or concerns the patient has before passing the prescription to be filled by a technician. This allows issues or concerns to be identified and addressed up front, rather than waiting until the prescription is filled and the patient is ready to leave the pharmacy. The primary outcome assessed in this study was change in blood pressure and/or A1C from baseline to the last follow-up visit. We reviewed 215 patient records. The mean age was 69.4 years (standard deviation 12.5), 51.2% of patients were male, 57.7% had hypertension, 5.6% had diabetes, and 36.7% had both. Median follow-up time was 4.2 months (interquartile range 2.5*-*9.3). In 203 patients with hypertension, systolic blood pressure was reduced from 139.83 mmHg to 131.26 mmHg (*p* < 0.001) and diastolic blood pressure from 80.26 mmHg to 76.86 mmHg (*p* < 0.001). In 87 patients with diabetes, A1C changed from 7.4% to 7.2% (*p* = ns). The P1st workflow model demonstrated significant improvements in blood pressure. Further investigation is needed to evaluate the effectiveness of this model with a control group, longer follow-up and evaluation of the patient experience.

## Introduction

Cardiovascular disease (CVD) is one of the leading causes of death in Canada.^1^ The majority of cases are caused by modifiable risk factors such as hypertension and diabetes.^2^ Hypertension is the most prevalent chronic disease, affecting 1 in 4 Canadian adults aged 20 to 79, and is the leading modifiable risk factor for premature morbidity and mortality worldwide.^3,4^ Diabetes affects approximately 1 in 7 Canadians.^5^ Current epidemiological trends indicate that new diagnoses of hypertension and diabetes continue to increase. Indeed, in 2018 it was projected that 1 in 3 Canadians would have either diabetes or prediabetes by 2020.^6^ As well, approximately 6 in 10 Canadians diagnosed with diabetes were also diagnosed with hypertension.^6^

Pharmacists are front-line primary care providers who see patients with hypertension and diabetes frequently. Numerous trials have demonstrated that pharmacist intervention significantly improves health outcomes, such as effective management of blood pressure and glycemic control and better medication adherence.^7-10^ Furthermore, the accessibility of pharmacist puts them in a prime position to systematically identify patients with poorly controlled cardiovascular risk factors and help in their management. However, there is a clear lack of implementation beyond this evidence. Barriers in the implementation of hypertension and diabetes care by pharmacists may be related to current workflows that are more suited to dispensing than to patient care.^11^

Carlene Oleksyn is a pharmacy owner in Spruce Grove, Alberta. She has developed an innovative workflow known as “Pharmacist First” (P1st), which focuses on immediate patient contact with the pharmacist. The pharmacist first assesses prescription appropriateness (both new prescriptions and refills), reviews the relevant laboratory tests, discusses chronic disease control and answers any questions or concerns the patient has before passing the prescription to be filled by a technician.^12^ In contrast to a model where the pharmacist interacts with the patient only at the end of the care process, immediate pharmacist-patient interaction allows for pharmacy technicians to begin processing and dispensing the medication while the patient is being assessed by the pharmacist. As such, clinical issues or concerns can be identified up front and solved during this process. This not only reduces wait time but also increases workflow efficiency while allowing pharmacists to build rapport, conduct their assessment and design a care plan. All pharmacists in these pharmacies have their additional prescribing authority, laboratory test authorization, and injection authorization, further increasing the type of patient care they are able to provide.

P1st allows for increased opportunities to proactively identify therapeutic issues, counsel patients and manage chronic conditions such as hypertension and diabetes. Additionally, this innovative workflow expands the provision of care through increased time and priority spent on assessments and patient education. As well, these consultations allow pharmacists to spend time with their patients to develop a comprehensive annual care plan (CACP) to help manage their chronic conditions, identify drug therapy problems and establish a monitoring and treatment plan.^13^

The main aim of this study was to evaluate changes in blood pressure and glycemic control in patients with hypertension and/or diabetes receiving care at a pharmacy using the P1st workflow model.

## Methods

We conducted a retrospective chart review of patients with hypertension and/or diabetes in 2 community pharmacies that use the P1st approach in the Greater Edmonton Region. We included any patient with hypertension and/or type 1 or type 2 diabetes who had received care using the P1st workflow model. We identified patients for the study by running a report through the pharmacy software (Kroll Pharmacy Management Solution) to identify patients with hypertension and/or diabetes who received a CACP between January 2014 and March 2020.^14^ We excluded patients with gestational diabetes and those who did not have any follow-up visits.

Data were collected from patient records within the Kroll system by a trained graduate student from the University of Alberta. Information collected included demographics (age, sex), patient assessment (blood pressure, diabetes type, duration, A1C) and pharmacist intervention (interventions made by the pharmacist, frequency of follow-up visits). Data were collected for baseline and all follow-up visits.

The primary outcome was the change in blood pressure (in those with hypertension) and the change in A1C (in those with diabetes) from baseline to the last recorded follow-up. The secondary outcome was the percentage of patients achieving their recommended blood pressure targets.

Descriptive statistics, including mean (standard deviation [SD]), median (interquartile range [IQR]) and frequency (proportion), were used to analyze demographic and clinical characteristics. The primary and secondary outcomes were analyzed using paired *t* test. Results were considered statistically significant when *p* < 0.05. Statistical analyses were performed using STATA software (version 16.1; StataCorp LLC, College Station, TX).

This study was approved by the University of Alberta Health Research Ethics Board. Waiver of consent was granted, as no direct contact or interaction with patients occurred and all identifiers were removed from the files before their review.

Data management was performed by the EPICORE Centre (www.epicore.ualberta.ca).^15^

## Results

We reviewed 217 patient records and included 215 in the study (2 were excluded because they did not receive any follow-up visits). The mean age was 69.4 years (SD 12.5), 51.2% were male, 57.7% had hypertension, 5.6% had diabetes and 36.7% had both (Table 1). All patients with diabetes had type 2 diabetes.

**Table 1:** Baseline patient demographics

	Pharmacy #1	Pharmacy #2	Total
Male, *n* (%)	58 (61.1)	52 (43.3)	110 (51.2)
Female, *n* (%)	37 (38.9)	68 (56.7)	105 (48.8)
Mean age in years (SD)	63.5 (10.3)	74.2 (12)	69.4 (12.5)
Hypertension only, *n* (%)	48 (22.3)	76 (35.3)	124 (57.7)
Diabetes only, *n* (%)	6 (2.8)	6 (2.8)	12 (5.6)
Both hypertension and diabetes, *n* (%)	41 (19.1)	38 (17.7)	79 (36.7)

The median time for the first follow-up visit was 4.2 months (IQR 2.5*-*9.3). The median overall follow-up duration was 19 months (IQR 10.4*-*29.8). The median number of follow-up visits per patient was 6 (IQR 4*-*11).

In the 201 patients with hypertension, systolic blood pressure was reduced from 139.83 mmHg at baseline to 131.26 mmHg (*p* < 0.001) at the most recent follow-up visit (Table 2). Diastolic blood pressure was reduced from 80.26 mmHg at baseline to 76.86 mmHg (*p* < 0.001) at the most recent follow-up (Table 2). In the 87 patients with diabetes, A1C levels changed from 7.37% to 7.22% (*p* = ns) (Table 2). Of the 79 patients with both hypertension and diabetes, 2 patients had measurements only for A1C (with no blood pressures recorded) and 4 patients had measurements only for blood pressure (with no A1C levels recorded).

**Table 2:** Blood pressure and A1C changes

	Patient type (*n*)	Baseline, mean (SD)	Last follow-up, mean (SD)
Systolic blood pressure, mmHg	With diabetes (77)	138.75 (17.78)	130.9* (11.34)
	Without diabetes (124)	140.53 (15.83)	131.48* (15.74)
	Overall (201)	139.83 (16.54)	131.26* (14.20)
Diastolic blood pressure, mmHg	With diabetes (77)	79.92 (10.37)	75.34* (9.28)
	Without diabetes (124)	80.47 (9.90)	77.96* (10.51)
	Overall (201)	80.26 (10.02)	76.86* (10.11)
A1C, %	With diabetes (87)	7.37 (1.43)	7.22 (1.29)

* Indicates statistical significance (*p* < 0.05) compared with baseline.

Of the 124 patients with hypertension only, 82.1% met the target systolic blood pressure, 93.5% met the target diastolic blood pressure and 78.9% reached both targets (Table 3). For the 77 patients with both hypertension and diabetes, 53.2% met the target systolic blood pressure, 77.9% met the target diastolic blood pressure and 46.7% reached both targets (Table 3).

**Table 3:** Patients reaching target blood pressure recommendations

	Hypertension only (*n* = 124)	Both hypertension and diabetes (*n* = 77)
Recommended systolic blood pressure, mmHg	140	130
Patients meeting systolic blood pressure targets, *n* (%)*	115 (82.1)	41 (53.2)
Recommended diastolic blood pressure, mmHg	90	80
Patients meeting diastolic blood pressure targets, *n* (%)*	116 (93.5)	60 (77.9)
Patients meeting systolic and diastolic blood pressure targets, *n* (%)*	97 (78.9)	36 (46.7)

* Values for the recommended blood pressure targets are taken from guidelines by Hypertension Canada.^16^

## Discussion

There is clear and strong evidence for the impact of pharmacist prescribing and care in patients with hypertension and diabetes.^7-9^ What is missing is an implementation strategy that applies this evidence in real-world practice. We found a significant reduction in systolic blood pressure (absolute difference 8.57 mmHg) and diastolic blood pressure (3.40 mmHg) in patients with hypertension receiving care at a pharmacy using the P1st workflow model. These are clinically important reductions in blood pressure, suggesting that the P1st model of care, applied in a real-world setting, could be effective in improving patient outcomes.

In patients with hypertension only, most met the systolic and diastolic targets, with about 79% meeting both targets set by Hypertension Canada.^16^ In patients with diabetes and hypertension, fewer met the systolic target (53%) and diastolic target (78%) and 48% achieved both targets.^16^

There was no significant reduction in A1C between baseline and the last follow-up visit (7.4% to 7.2%). However, depending on the functionality of patients, the A1C reported could actually be considered at target. According to Diabetes Canada, the recommended target ranges for A1C for adults older than 65 years are between 7.1% and 8.0% for functionally dependent adults and ≤7.0% for functionally independent adults.^17^ Considering the average age of the patient population and the A1C recommendation from Diabetes Canada, this could explain the observed nonsignificant trend. Furthermore, glycemic control in this group was already quite good, leaving little room for improvement. This also highlights that patients with poorer glycemic control should be targeted for this service.

Our findings are consistent with those of the R_x_EACH study, a randomized trial which demonstrated that pharmacist intervention (assessment, education, prescribing and regular follow-up) in patients at high risk for CVD was associated with significant reduction in blood pressure, A1C and estimated cardiovascular risk.^7^ R_x_EACH demonstrated that a proactive pharmacist approach that allowed patients to spend more time with their pharmacists was much more successful in managing CVD risk factors than usual care.^7^

The findings from our study are also consistent with the findings from Santschi et al.,^9^ who conducted a systematic review of 39 randomized controlled trials and found that pharmacist intervention significantly reduced blood pressure by 7.6/3.9 mmHg compared with usual care.^9^

The P1st model is an implementation strategy that appears to produce results consistent with evidence in the literature that indicates proactive, pharmacist-led interventions are successful in managing CVD and its risk factors.^7,9,18,19^ Indeed, when pharmacists are able to practise to their full scope (assessing patients, prescribing, administering injections, ordering and interpreting laboratory tests and providing disease management), better outcomes have been reported for patients with chronic conditions.^7,20,21^

There are a number of limitations to the current study. The lack of randomization and a control group makes it difficult to determine a causal relationship. There was no standardized measurement of blood pressure or a prespecified follow-up schedule. Measurements recorded from patient records could be from the patient’s home, a physician’s office or the pharmacy. Our follow-up duration was relatively short, and as a retrospective chart review, documentation was sometimes limited. We were not able to capture components of pharmacist interventions due to limited documentation and, as a result, we could not determine that pharmacists were practising to their full scope. Although we examined 2 independent community pharmacies for this study, it is possible that the effects observed are simply due to the exceptional pharmacists themselves. Furthermore, patient selection was based on having a CACP, and therefore it is not clear whether changes are a result of the P1st workflow model or the care plan itself. Nevertheless, our study provides promising evidence for a new, proactive model of care that should be investigated further.

Further research should compare the P1st model to a traditional workflow model in a randomized controlled trial, examining multiple community pharmacies, with standardized follow-up, analysis of the patient journal and consistent documentation of patients’ health measurements throughout the study. This would help us better evaluate this innovative workflow model and establish whether it is more effective (and cost-effective) than traditional workflow models in improving patient outcomes.

## Conclusion

As the health care professionals who see patients with chronic diseases the most frequently, pharmacists are well positioned to provide care and help patients achieve their health goals. In this real-world study, we observed a significant reduction in systolic and diastolic blood pressure for patients being treated under the P1st workflow model. Adapting this model of care has the potential to significantly improve patient health outcomes. ■

## References

[1] Public Health Agency of Canada. Report from the Canadian Chronic Disease Surveillance System: Heart Disease in Canada, 2018. Available: https://www.canada.ca/en/public-health/services/publications/diseases-conditions/report-heart-disease-Canada-2018.html#s6 (accessed April 17, 2021).

[2] American Diabetes Association. *Cardiovascular disease and risk management.* Sec. 9. In Standards of Medical Care in Diabetes 2017. Diabetes Care 2017;40(Suppl. 1):S75-S87. 10.2337/dc17-S012 27979896

[3] Padwal RS Bienek A McAllister FA Campbell NR. Epidemiology of hypertension in Canada: an update. Can J Cardiol 2016;32(5):687-94. 10.1016/j.cjca.2015.07.734 26711315

[4] Mills KT Bundy JD Kelly TN , et al. Global disparities of hypertension prevalence and control: a systematic analysis of population-based studies from 90 countries. Circulation 2016;134(6):441-50. 10.1161/CIRCULATIONAHA.115.018912 PMC4979614 27502908

[5] Government of Canada SC. Diabetes, 2017. Available: https://www150.statcan.gc.ca/n1/pub/82-625-x/2018001/article/54982-eng.htm (accessed Sept. 4, 2020).

[6] Weisman A Fazli GS Johns A Booth GL. Evolving trends in the epidemiology, risk factors and prevention of type 2 diabetes: a review. Can J Cardiol 2018;34(5):552-64. 10.1016/j.cjca.2018.03.002 29731019

[7] Tsuyuki RT Al Hamarneh YN Jones CA Hemmelgarn BR. The effectiveness of pharmacist interventions on cardiovascular risk: the multicenter randomized controlled R_x_EACH Trial. J Am Coll Cardiol 2016;67:2846-54. 10.1016/j.jacc.2016.03.528 27058907

[8] Wubben DP Vivian EM. Effects of pharmacist outpatient interventions on adults with diabetes mellitus: a systematic review. Pharmacotherapy 2008;28:421-36. 10.1592/phco.28.4.421 18363526

[9] Santschi V Chiolero A Colosimo AL , et al. Improving blood pressure control through pharmacist interventions: a meta-analysis of randomized controlled trials. J Am Heart Assoc 2014;3:e000718. 24721801 10.1161/JAHA.113.000718 PMC4187511

[10] Milosavljevic A Aspden T Harrison J. Community pharmacist-led interventions and their impact on patients’ medication adherence and other health outcomes: a systematic review. Int J Pharm Pract 2018:26(5):387-97. 10.1111/ijpp.12462 29927005

[11] Goode J-V Owen J Page A Gatewood S . Community-based pharmacy practice innovation and the role of the community-based pharmacist practitioner in the United States. Pharmacy (Basel) [Internet]. 2019 Aug 4 [accessed 2020 Sept. 7];7(3). Available from: https://www.ncbi.nlm.nih.gov/pmc/articles/PMC6789634/10.3390/pharmacy7030106 PMC6789634 31382690

[12] Alberta College of Pharmacy. The future is now. Available: https://abpharmacy.ca/articles/future-now (accessed Aug. 1, 2020).

[13] Alberta Blue Cross. Compensation for pharmacy services. Pharmacy Benefact. 2012;346:4.

[14] TELUS Health. Kroll™ pharmacy management solution. Available: https://www.telus.com/en/health/health-professionals/pharmacies/kroll (accessed Aug. 1, 2020).

[15] EPICORE Centre. Available: https://www.epicore.ualberta.ca/home/ (accessed Aug. 2, 2020).

[16] Nerenberg KA Zarnke KB Leung AA , et al. Hypertension Canada’s 2018 guidelines for diagnosis, risk assessment, prevention and treatment of hypertension in adults and children. Can J Cardiol 2018;34(5):506-25. 10.1016/j.cjca.2018.02.022 29731013

[17] Meneilly GS Knip A Miller DB Sherifali D Tessier D Zahedi A. Diabetes in older people. Can J Diabetes 2018;42:S283-95. 10.1016/j.jcjd.2017.10.021 29650107

[18] Houle SKD Chuck AW McAlister FA Tsuyuki RT . Effect of a pharmacist-managed hypertension program on health system costs: an evaluation of the Study of Cardiovascular Risk Intervention by Pharmacists-Hypertension (SCRIP-HTN). Pharmacotherapy 2012;32(6):527-37. 10.1002/j.1875-9114.2012.01097.x 22552863

[19] Tsuyuki RT Houle SKD Charrois TL , et al. Randomized trial of the effect of pharmacist prescribing on improving blood pressure in the community: the Alberta Clinical Trial in Optimizing Hypertension (R_x_ACTION). Circulation 2015;132(2):93-100. 26063762 10.1161/CIRCULATIONAHA.115.015464

[20] The Conference Board of Canada. The value of expanded pharmacy services in Canada. Available: https://www.conferenceboard.ca/e-library/abstract.aspx?did=8721&AspxAutoDetectCookieSupport=1 (accessed Jul. 25, 2020).

[21] Hamarneh YNA Charrois T Lewanczuk R Tsuyuki RT . Pharmacist intervention for glycaemic control in the community (the RxING study). BMJ Open 2013;3(9):e003154. 10.1136/bmjopen-2013-003154 PMC3787489 24068762

